# Mechanisms of CAR T cell exhaustion and current counteraction strategies

**DOI:** 10.3389/fcell.2022.1034257

**Published:** 2022-12-08

**Authors:** Xiaoying Zhu, Qing Li, Xiaojian Zhu

**Affiliations:** ^1^ Department of Hematology, Tongji Hospital, Tongji Medical College, Huazhong University of Science and Technology, Wuhan, China; ^2^ Department of Hematology, Wuhan No. 1 Hospital, Wuhan, China

**Keywords:** CAR T cell exhaustion, tonic signaling, cytokines, tumor microenvironment, immune checkpoint blockade, epigenetics, transcriptomics

## Abstract

The functional state of chimeric antigen receptor T (CAR T) cells determines their efficacy *in vivo*. Exhausted CAR T cells exhibit decreased proliferative capacity, impaired anti-tumor activity, and attenuated persistence. CAR T cell exhaustion has been recognized as a vital cause of nonresponse and relapse after CAR T cell therapy. However, the triggers and mechanisms leading to CAR T cell exhaustion remain blurry and complicated. Therefore, it is essential to clear the regulation network of CAR T cell exhaustion and explore potent solutions. Here, we review the diverse inducers of CAR T cell exhaustion in terms of manufacture process and immunosuppressive tumor microenvironment. In addition to the admitted immune checkpoint blockade, we also describe promising strategies that may reverse CAR T cell exhaustion including targeting the tumor microenvironment, epigenetics and transcriptomics.

## Introduction

Despite the tremendous efficacy of chimeric antigen receptor T (CAR T) cells in hematological malignancies such as B-cell acute lymphoblastic leukemia (B-ALL), B-cell lymphoma, and multiple myeloma, as well as in solid tumors, CAR T cell exhaustion remained a main obstacle to achieve remission (Fraietta et al., 2018; Holstein and Lunning, 2019; Marofi et al., 2021). T cell exhaustion was a dysfunctional state of T cells that usually observed in chronic infections and tumors (Hashimoto et al., 2018). It was first identified as a population of CD8^+^ T cells without effector function which appeared during chronic lymphocytic choriomeningitis virus infection in mice, and the persistence of this cell population led to viral immune escape (Zajac et al., 1998). At the same time, Gallimore and colleagues also observed a similar phenomenon in the chronic lymphocytic choriomeningitis virus infection, which they defined as T cell exhaustion (Gallimore et al., 1998). T cell exhaustion was characterized by progressive loss of effector function, co-expression of multiple inhibitory receptors, and exaggerated effector cell differentiation. Besides, the exhausted T cells had distinct transcriptional, epigenetic, and metabolic signatures (Doering et al., 2012; Schietinger and Greenberg, 2014; Philip et al., 2017).

Abundant clinical practice and research revealed that CAR T cell exhaustion ultimately contributed to the failure of CAR T cell therapy. For instance, Fraietta et al. performed transcriptomic sequencing and functional assessment of CAR T cells from 41 patients with chronic lymphocytic leukemia (CLL). They identified that CAR T cells in responders possessed memory-like characteristics, while CAR T cells in non-responders were in a highly exhaustion state (Fraietta et al., 2018). Consistent with this, other clinical studies on B-ALL and B-cell lymphoma also indicated that CAR T cell exhaustion was related to the treatment failure (Schuster et al., 2017; Finney et al., 2019; Deng et al., 2020). Therefore, the explorations focused on the mechanisms of CAR T cell exhaustion may provide potential insights on preventing CAR T cell exhaustion and improving CAR T cell efficacy.

In this article, we reviewed the underlying regulatory factors of CAR T cell exhaustion that existed in design, generation and infusion process. We also discussed the current strategies to combat CAR T cell exhaustion including blocking immune checkpoint, resisting the tumor microenvironment (TME), and regulating epigenetic and transcriptomic profiles.

## CAR design affects CAR T cell exhaustion

**FIGURE 1:**
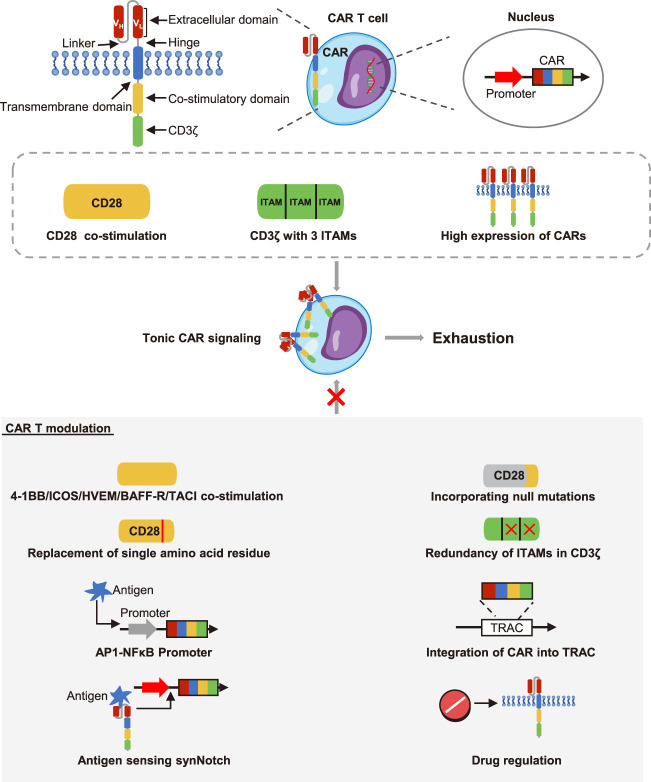
The effects of CARs structure on CAR T cell exhaustion. The CARs contained an extracellular domain, a hinge region, a transmembrane domain, a co-stimulatory domain and a CD3ζ stimulatory domain. The extracellular domain was composed of the variable heavy chain (V_H_) and the variable light chain (V_L_) connected by a linker. The upstream promoter initiated the strong expression of CARs on the cell surface. However, the CD3ζ with 3 immunoreceptor tyrosine-based activation motifs (ITAMs), CD28 co-stimulation and constitutively high expression of CARs could cause tonic CAR signaling and promote cell exhaustion. However, the 4-1BB, ICOS, HVEM, BAFF-R, and TACI co-stimulation reduced the exhaustion of CAR T cells. Both incorporation of null mutations and replacement of single amino acid residue in CD28 co-stimulation could inhibit exhaustion. Redundancy of ITAMs could also inhibit exhaustion. Besides, the synthetic activator protein 1-nuclear factor κB (AP1-NF-κB) promoter, the T cell receptor alpha constant locus (TRAC) that contained CAR structure, the synNotch-CAR circuits, and drug regulation could all restrain the CARs expression. Then the ligand-independent tonic signaling and exhaustion were thus reduced.

The second generation of CARs approved by the US Food and Drug Administration contained an antigen binding domain, a hinge region, a transmembrane domain, an intracellular co-stimulatory domain and a signal transduction domain (Zhao et al., 2018). CAR molecules were activated upon stimulation by an antigen, which triggered the effector response (Salter et al., 2021). Nevertheless, undesirable CAR design would lead to suboptimal T cell activation and ligand-independent tonic signaling (Jayaraman et al., 2020). Long and colleagues described that GD2, CD22, or ErbB2 CAR T cells contained a CD28-CD3ζ intracellular domain tended to be exhausted during early expansion *in vitro*, but CD19.28z CAR T cells did not. This was due to the tonic CAR signaling induced by aggregation of single-chain variable fragments (scFv) on the surface (Figure 1). The co-stimulatory domain was also involved in tonic CAR signaling, with CD28 co-stimulation enhancing while 4-1BB co-stimulation reducing the tonic CAR signaling and exhaustion (Long et al., 2015). Intriguingly, it was proved that introduction of null mutations in the CD28 domain would down-regulate exhaustion-related genes and improve the anti-tumor function of CD19 CAR T cells *in vitro* and *in vivo* (Boucher et al., 2021). Similarly, ICOS co-stimulation also had an advantage over CD28 co-stimulation in averting exhaustion. Indeed, CD28 co-stimulation and ICOS co-stimulation had a shared motif but differed only in a single amino acid residue. Guedan et al. observed that this single amino acid residue asparagine in CD28 was related to the exhaustion of CAR T cells, and replacing asparagine with phenylalanine reduced CAR T cell differentiation and exhaustion in xenograft tumor models (Guedan et al., 2020). Moreover, Nunoya and colleagues evaluated the feature of CAR T cells with different co-stimulatory domain *in vitro*, and they identified that the co-stimulatory domain derived from herpes virus entry mediator (HVEM) was favorable for a low-level of exhaustion (Nunoya et al., 2019). More recently, a report using CAR pooling to evaluate the propensity to resist exhaustion of CAR T cells during repetitive antigen stimulation. It was revealed that B cell-activating factor receptor (BAFF-R) and transmembrane activator and CAML interactor (TACI) delayed the exhaustion (Goodman et al., 2022). In addition to the co-stimulatory domain, signaling of the intracellular domain of CD3ζ also affected CAR T cell exhaustion. Using the xenograft model, Feucht et al. showed CD19 CAR T cells retaining only a single membrane-proximal immunoreceptor tyrosine-based activation motif (ITAM) had lower exhaustion levels and stronger persistence compared with conventional CAR T cells that had 3 ITAMs (Feucht et al., 2019).

Another important factor to consider when designing CAR T cells was the expression level of the CARs. In fact, the expression of T cell receptors (TCRs) was strictly controlled by the CD45 molecules and co-inhibitory receptors after initiating activation, but CARs typically contained a strong constitutive promoter to ensure their long-term and stable expression in T cells (Harris and Kranz, 2016; Wu et al., 2020). However, it had been recently reported that high expression of CAR molecules acted as a critical inducer of tonic CAR signaling and exhaustion (Figure 1). To disrupt constitutively expressed CARs, Webster and colleagues constructed the synthetic activator protein 1 (AP1)-nuclear factor κB (NFκB) promoter, which reduced the CAR expression at rest and drove the CAR expression under antigen stimulation. Consequently, AP1-NFκB promoted CAR T cells showed limited ligand-independent tonic signaling and exhaustion in preclinical models (Webster et al., 2021). In another attempt, Eyquem et al. designed CAR T cells through integrating the CD19 CAR into the T cell receptor alpha constant locus. These CAR T cells enabled CAR molecules to be low expressed but dynamically regulated under tumor antigen stimulation, thereby they could control tonic CAR signaling in the absence of antigen and avoid exhaustion *in vitro* (Eyquem et al., 2017). Recently, T cells with synthetic Notch (synNotch) receptors were developed to improve the specificity of CAR T cells. These CAR T cells with synNotch-CAR circuits activated the CAR expression only when they recognized the synNotch antigen (Srivastava et al., 2019). Impressively, this structure was showed to effectively prevent tonic signaling and enable CAR T cells to maintain memory phenotype and resist exhaustion in mouse models (Hyrenius-Wittsten et al., 2021). Besides, Weber et al. demonstrated that incorporating an FK506 binding protein 12 destabilizing domain into GD2 CAR structure would make the expression of CAR subject to the drug shield-1, this drug-regulatable system reversed the exhausted state of CAR T cells *in vitro* and in preclinical models. They also described that dasatinib which had been reported to reversibly inhibit TCR and CAR signaling had similar effects in reversing CAR T cells exhaustion (Weber et al., 2021). This study provided an extremely promising example for modulating the activation of CAR T cells to control their efficacy and exhaustion in clinical practice. The pharmacologic on/off switch may become a viable approach to steer the functional state of CAR T cells *in vivo*. Of note, Huang et al. initiated a clinical research (NCT04603872) to investigate the combination effects of dasatinib on *in vitro* manufacturing and *in vivo* infusion of CAR T cells.

Overall, the structure of CAR T cells was closely related to their functional characteristics. The component elements including scFv molecules, costimulatory domains and intracellular signaling domains all had complex regulatory effects on the activation and exhaustion. Optimizing the structure and regulating the expression pattern of CARs were feasible to weaken the tonic CAR signaling and prevent the exhaustion.

## The *in vitro* expansion condition affects CAR T cell exhaustion

**FIGURE 2:**
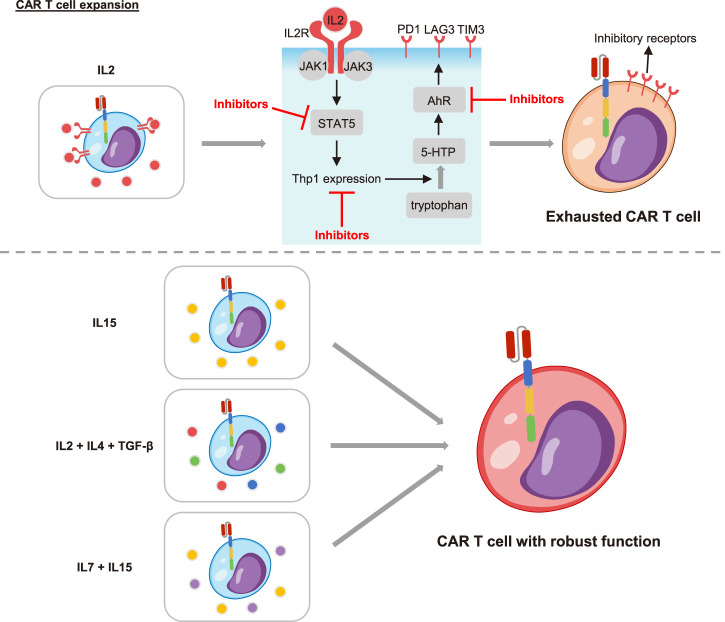
The effects of cytokines on CAR T cell exhaustion. The cytokine IL2 promoted CAR T cell exhaustion through the IL2 receptor (IL2R). The combination of IL2 and IL2R led to the recruitment and activation of the Janus family of tyrosine kinases (JAK1 and JAK3). Then the signal transducer and activator of transcription 5 (STAT5) was activated and further induced the expression of tryptophan hydroxylase 1 (Tph1). The Thp1 catalyzed tryptophan to produce 5-hydroxytryptophan (5-HTP), which activated the aryl hydrocarbon receptor (AhR) and led to T cell exhaustion. Inhibitors of STAT5, Thp1 or AhR could all restrain T cell exhaustion. Importantly, the cytokine IL15, IL2 supplemented with IL4/TGF-β, and IL15/IL7 improved the exhaustion. CAR T cells under these culture conditions manifested enhanced persistence and function.

In order to obtain sufficient CAR T cell numbers, CAR T cells needed to be expanded *in vitro* before infused into the patient. There was a growing body of evidence that the culture system also affected the exhaustion of CAR T cells (Figure 2). The cytokine IL2, which was commonly added to the expansion system would cause CAR T cell exhaustion and reduce their *in vivo* persistence. However, CAR T cells expanded under cytokine IL15 or IL2 plus IL4 and TGF-β maintained a less differentiated phenotype. They exhibited reduced expression of inhibitory receptors and strengthened antitumor efficacy *in vitro* and *in vivo* (Gattinoni et al., 2005; Alizadeh et al., 2019; Liu et al., 2020). Consistently, Giuffrida et al. described that IL15 promoted the production of central memory T cells and up-regulated memory-related genes including TCF7. The persistence and response to adjuvant anti-PD1 therapy were thus significantly enhanced in preclinical models (Giuffrida et al., 2020). Similarly, addition of other cytokines such as IL4, IL7 and IL21 also supported the stemness of CAR T cells and restrained the exhaustion (Ptáčková et al., 2018). Based on these, the clinical research to ascertain the efficacy of IL15 plus IL7 and IL15 plus IL21 were initiated (NCT04186520, NCT04715191). Moreover, it was recently reported that the exhaustion level of CAR T cells increased with the prolongation of expansion time *in vitro*. The “younger” cells with shorter culture time manifested lowered exhaustion markers and enhanced long-term killing function *in vivo* (Caruso et al., 2019). In summary, the *in vitro* expansion condition greatly impacted the differentiation and exhaustion transition of CAR T cells. It was extremely important to modulate the cytokines and expansion time to obtain CAR T cells with optimal phenotypes and healthy states.

## The TME promotes CAR T cell exhaustion

**FIGURE 3:**
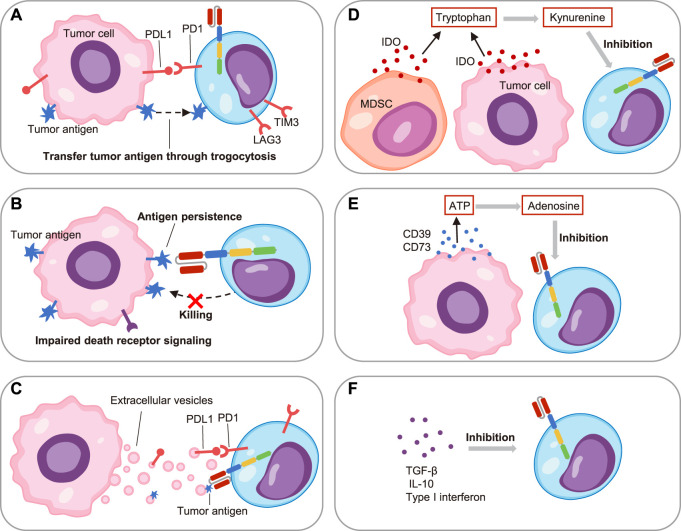
The factors promoting CAR T cell exhaustion in the tumor microenvironment.**(A)** On one hand, the PDL1 on tumor cells induced CAR T cell exhaustion through PD1/PDL1 pathway; on the other hand, the tumor antigen transferred to CAR T cells through trogocytosis and caused exhaustion. **(B)** The impaired death receptor signaling of leukemia cells led to their resistance to CAR T cells, and the persistent antigen in turn led to CAR T cell exhaustion. **(C)** The extracellular vesicles secreted by leukemia cells induced CAR T cell exhaustion through PDL1 and tumor antigen. **(D)** The myeloid-derived suppressor cells (MDSC) and leukemia cells secreted indoleamine 2,3-dioxygenase (IDO), which catalyzed tryptophan to kynurenine to suppress CAR T cell function. **(E)** The CD39 and CD73 released by tumor cells hydrolyzed ATP to adenosine, which inhibited CAR T cell activity. **(F)** Soluble factors including TGF-β, IL-10 and type I interferon restrained CAR T cell activity.

Recently, it was reported that the TME promoted by malignant cells played an important role in tumor development and immune regulation (DeBerardinis, 2020). The hallmarks of the TME including diverse cells such as tumor cells, immune cells and stromal cells, as well as soluble factors such as cytokines, metabolites and extracellular vesicles (EVs) (Anderson and Simon, 2020). These complex components exerted intricate regulatory effects on CAR T cells (Figure 3).

As the pivot, tumor cells induced CAR T cell exhaustion versatilely. For example, the multiple immune checkpoint ligands such as PDL1 on tumor cells interacted with their corresponding receptors on CAR T cells and resulted in cell exhaustion (Wang et al., 2019). Additionally, it was reported that the targeted antigen on the ALL cells would be transferred to CAR T cells through trogocytosis in xenograft mouse models, which promoted the expression of PD1, LAG3 and TIM3. Intriguingly, this phenomenon also caused the fratricide of CAR T cells (Hamieh et al., 2019). More recently, Singh et al. showed the impaired death receptor signaling in ALL cells caused the resistance to cytotoxicity of T cells, and thus the persistence of antigens induced functional impairment of CAR T cells in turn. Impressively, they observed that the inherent dysregulation of death receptor signaling in ALL cells directly contributed to CAR T cell exhaustion and correlated with clinical outcomes (Singh et al., 2020). Although CD19 CAR T cells were applied to treat CLL, B-ALL, and B-cell lymphoma, they were less effective in CLL. An explanation for this discrepancy was that the imbalance between the suppressive TME and immune cells (Yoon et al., 2018). Jitschin and colleagues observed that the myeloid-derived suppressor cells (MDSC) were significantly increased in the peripheral blood of CLL patients. On one hand, this MDSC subset promoted the regulatory T cells (Treg). On the other hand, MDSC secreted high levels of indoleamine 2, 3-dioxygenase (IDO) to metabolize tryptophan to limit T cell proliferation and function (Jitschin et al., 2014). All in one, these findings highlighted the significance of the immunosuppressive cell components for inducing CAR T cell exhaustion.

As for immunosuppressive soluble factors in the TME, EVs have attracted the most attention. Cox et al. identified that the number of EVs in patients with CLL was significantly higher than the number in normal individuals, and the same was true for PDL1 positive EVs. They further verified that PDL1 positive EVs induced CD19 CAR T cell exhaustion and attenuated effector function *in vitro* and *in vivo* (Cox et al., 2021). Our recent studies also demonstrated that EVs secreted by tumor cells such as B-ALL could induce CD19 CAR T cell exhaustion. Mechanistically, we found that B-ALL cells secreted EVs specifically carrying CD19 antigen, and thus their persistent antigenic stimulation led to exhaustion of CD19 CAR T cells (Zhu et al., 2022). In addition to EVs, tumor cells also secreted masses of metabolites to tame the TME, and consequently to regulate immunity. For instance, the AML cells in bone marrow and B-cell lymphoma cells released IDO to degrade tryptophan, resulting in local nutrient depletion while producing metabolites such as kynurenine to inhibit CAR T cell activity (Teague and Kline, 2013; Ninomiya et al., 2015). Recently, Mussai and colleagues described the AML blast in patients generated Arginase II, which consumed the arginine and led to low-arginine microenvironment. It was proved that the low-arginine conditions would cause the exhaustion and defective proliferation of CAR T cells *in vitro* (Mussai et al., 2019). Similarly, tumor cells produced ectoenzymes CD39 and CD73, which would hydrolyze ATP to generate adenosine. The adenosine further bound to adenosine 2A receptors on T cells to inhibit their function (Leone et al., 2015; Beavis et al., 2017). In addition, other soluble factors such as transforming growth factor-β (TGF-β), IL-10 and type I interferon were also important regulators of CAR T cell function in the TME (Chang et al., 2018; Stüber et al., 2019). Overall, a great deal of efforts had been devoted to the immune regulation of TME. However, the TME was extremely complex, new drivers of CAR T cell exhaustion were constantly emerging.

## Strategies for combating CAR T cell exhaustion

### Immune checkpoint blockade

An important hallmark of CAR T cell exhaustion was the up-regulation of multiple inhibitory receptors such as PD1, CTLA4, and LAG3. Their binding to corresponding ligands on tumor cells promoted the dysfunction of exhausted T cells and led to tumor immune escape. Cherkassky et al. proved that PD1-mediated cell exhaustion significantly inhibited CAR T cell function. Blocking the PD1-PDL1 pathway by PD1 inhibitors or endogenous blockade of PD1 expression could resist exhaustion and enhance function *in vitro* and in preclinical animal models (Cherkassky et al., 2016). Therefore, PD1 and CTLA4 inhibitors aimed at disrupting the inhibitory receptor pathway had gradually been applied for improving tumor immunity in clinical practice (Kon and Benhar, 2019; Liu, 2019). Cao et al. evaluated the efficacy of combination of CD19 CAR T cells with anti-PD1 antibody (nivolumab) in patients with refractory/relapsed B-cell non-Hodgkin lymphoma (Table 1). Encouragingly, they observed that complete response could be achieved in 45.45% of patients (Cao et al., 2019). Similarly, one recent study reported that the PD1 inhibitor, pembrolizumab, achieved complete remission or partial remission in 25% of patients with B-cell lymphoma refractory to and/or relapsed after CD19 CAR T cells treatment. Immune analysis revealed the patients who achieved remission had mild CAR T cell exhaustion, which indicating that PD1 inhibitors had certain benefits in improving exhaustion and enhancing the efficacy of CD19 CAR T cells (Chong et al., 2022). Meanwhile, administration of CAR T cells with nivolumab and ipilimumab (CTLA-4 inhibitor) was also being tested in clinical trials (NCT04003649).

**TABLE 1:** Strategies for combating CAR T cell exhaustion.

Strategies	Targets	References
Anti-PD1 antibody, CTLA-4 inhibitor	PD1-PDL1,CTLA-4	(Cao et al., 2019; Chong et al., 2022),NCT04003649
Soluble PD1-CH3 fusion protein	PD1-PDL1	Pan et al. (2018)
Downregulating immune checkpoint	TIGIT, PD1,TIM3, LAG3	Zou et al. (2019); Lee et al. (2022)
A2AR knockout, A2AR antagonists,ADA-overexpression	Adenosine	Beavis et al. (2017); Li et al. (2020); Qu et al. (2022)
ASS/OTC expression, Inhibiting arginine metabolism	Low-arginine	Mussai et al. (2019); Fultang et al. (2020)
DnTGF-βRII co-expression,TGF-βRII knockout, Bispecific trap protein	TGF-β	Kloss et al. (2018); Tang et al. (2020); Chen et al. (2021)
CD4 CAR T cell, IL7 co-expression		Agarwal et al. (2020); He et al. (2020)
DNMT3A knockout, Decitabine	Remodeled methylation programs	You et al. (2020); Wang et al. (2021b); Prinzing et al. (2021)
		NCT04697940
		NCT04850560
		NCT04553393
BET protein blockade	CAR silencing	Kong et al. (2021)
NR4A triple knockout	NR4A TFs	Chen et al. (2019)
TOX and TOX2 knockout	TOX and TOX2	Seo et al. (2019)
c-Jun-overexpression	AP1 TFs	Lynn et al. (2019)
Inhibiting HPK1	HPK1	Si et al. (2020)
TLE2 and IKZF2 knockout	TLE2 and IKZF2	Wang et al. (2021a)

In addition to PD1 and CTLA4 inhibitors, modifying CAR T cells to block immune checkpoint pathways had also been investigated intensively. For example, Pan and his colleagues introduced a fusion protein consisting of the extracellular domain of PD1 and CH3 from IgG4 into GPC3 CAR T cells to construct CAR T cells secreting a soluble PD1 protein. They found these CAR T cells were protected from exhaustion when stimulated by target cells *in vitro* and in murine xenogeneic models (Pan et al., 2018). Furthermore, Zou et al. demonstrated simultaneously downregulating three checkpoint receptors PD1, TIM3, and LAG3 could improve the exhaustion of CAR T cells through upregulation of CD56 in xenogenic mouse models (Zou et al., 2019). In line with this, a recent study described that simultaneous down-regulation of PD1 and TIGIT exerted a synergistic anti-tumor effect, the down-regulation of TIGIT was mainly responsible for maintaining the low-differentiation and low-exhaustion state of CAR T cells, while the down-regulation of PD1 enhanced the short-term cytotoxicity of CAR T cells *in vitro* functional assays (Lee et al., 2022). In summary, immune checkpoint blockade had achieved certain successes in preclinical and clinical practice, which was also the mainstream method for resisting CAR T cell exhaustion currently.

### Combating the TME

With the clarification of the complex role of TME, new strategies targeting TME to improve the efficacy of CAR T cells had proliferated recently (Table 1). In terms of the excessive adenosine in the TME, the adenosine 2A receptor on CAR T cells was responsible for mediating its immunosuppressive effects. Accordingly, it was reported that using the adenosine 2A receptor antagonists and knockout of receptors in CAR T cells improved exhaustion and function in preclinical mouse models (Beavis et al., 2017; Li et al., 2020). Besides, to confront adenosine, immune cells could also catabolize it into inosine through adenosine deaminase 1 (ADA). Therefore, Qu et al. engineered ADA-overexpressing CAR T cells and proved that they had an enhanced ability to resist exhaustion *in vitro* and *in vivo* (Qu et al., 2022).

As for the arginine, it was recognized that endogenous production of arginine was mainly dependent on argininosuccinate synthetase (ASS) enzymes and ornithine transcarbamylase (OTC). However, the expression of ASS and OTC in T cells was low, so they were dependent on exogenous arginine and were sensitive to low-arginine microenvironment. Encouragingly, inhibiting the arginine metabolism or engineering CAR T cells to express functional ASS and OTC could both significantly enhance the function (Mussai et al., 2019; Fultang et al., 2020).

To overcome the excess TGF-β in the TME, Kloss et al. blocked TGF-β signaling by co-expressing a dominant-negative TGF-β receptor II (TGF-βRII) in PSMA CAR T cells targeting prostate cancer. Tang et al. used CRISPR/Cas9 technology to knockout endogenous TGF-βRII in CAR T cells. Both these modifications prevented CAR T cell exhaustion and enhanced anti-tumor effects in tumor models (Kloss et al., 2018; Tang et al., 2020). And there were abundant agents that were being tested in clinical trials including TGF-β targeted neutralizing antibodies, vaccines, antisense oligonucleotides, and small molecule inhibitors. Of particular interest, a mass of research devoted to developing agents that dual-targeting TGF-β and PDL1 molecules. Especially the bintrafusp alfa, a bifunctional fusion protein targeting TGF-β and PD-L1 (Gulley et al., 2022). More recently, Chen and his team designed CAR T cells with a bispecific trap protein structure that secreted trap proteins, which could simultaneously target TGF-β and PD1. Such CAR T cells significantly attenuated suppressive T cell signaling and resisted exhaustion *in vitro* and *in vivo* (Chen et al., 2021).

Furthermore, in order to clear the ability of different types of CAR T cells to resist exhaustion in the TME, one study used CD4 or CD8-targeted lentivirus to generate CD4 CAR T cells or CD8 CAR T cells *in vivo*, respectively. It was showed that the anti-tumor efficacy of CD4 CAR T cells alone was better than that of CD8 CAR T cells alone or mixture of CD4 and CD8 CAR T cells, because CD8 CAR T cells were more prone to exhaustion under high tumor burden. This report suggested that the phenotype could be adjusted to obtain CAR T cells that were resistant to exhaustion *in vivo* (Agarwal et al., 2020). In addition, promoting the secretion of cytokines could also resist CAR T cell exhaustion caused by the TME. For example, co-expression of IL7 gene enabled CAR T cells to produce IL7 under antigen stimulation, which promoted CAR T cell proliferation and inhibited CAR T cell exhaustion and apoptosis. These CAR T cells exhibited a less differentiated phenotype with a higher proportion of central memory T cells in preclinical models (He et al., 2020). Therefore, it was feasible to engineer CAR T cells that directly counter the immune suppressors in the TME to resist exhaustion. However, the clinical efficacy and safety of these CAR T cells remained to be further observed.

### Epigenetic regulation of CAR T cell exhaustion

It had been established that exhausted T cells had completely different epigenetic characteristics from effector T cells and memory T cells. For instance, exhausted T cells lacked several open chromatin regions presented in the IFNG locus in effector T cells and memory T cells. Because blocking PD1 was unable to fully remodel the epigenetics of exhausted T cells, T cells would be re-exhausted when the antigen persisted (Pauken et al., 2016). Subsequently, Ghoneim et al. performed whole-genome sequencing of effector T cells and exhausted T cells, they identified that the acquired *de novo* methylation programs limited T cell proliferation and clonal diversity during transition to exhaustion (Ghoneim et al., 2017). In line with this, Zebley and colleagues observed CAR T cells acquired exhaustion-related methylation programs after infusion in patients with relapsed/refractory B-ALL, the genes associated with T cell memory maintenance including TCF and LEF1 were suppressed (Zebley et al., 2021). Consequently, restraining these epigenetic modifications had the potential to reverse exhaustion transition (Table 1). In a recent work, it was described that blocking *de novo* methylation programs through *de novo* DNA methyltransferase 3α (DNMT3A) knockout could overcome CAR T cell exhaustion *in vitro* and in preclinical solid tumor models (Prinzing et al., 2021). Similarly, DNA methyltransferase inhibitor, decitabine, had also been proved to reverse exhaustion-associated DNA-methylation programs. Two research teams successively demonstrated that decitabine enhanced the antitumor activity of CAR T cells both *in vitro* and *in vivo*. Through transcriptome and epigenetic sequencing, it was revealed that memory-related genes and immune synapse genes were promoted while exhaustion-related genes were suppressed (You et al., 2020; Wang Y. et al., 2021b). In addition, clinical trials of CD19 CAR T cells in relapsed/refractory CLL (NCT01029366 and NCT01747486) showed the CAR silencing was associated with CAR T treatment failure, this was caused by abnormal methylation programs of the CAR promoter. Kong and colleagues presented that epigenetic modulation by bromodomain and extra-terminal (BET) family protein blockade could reverse CAR silencing and improve CAR T cell exhaustion *in vitro* (Kong et al., 2021). Overall, these clinical and preclinical data provided evidence for the epigenetic involvement in the regulation of CAR T cell exhaustion and efficacy. In particular, the combination of decitabine with CAR T cell therapy was currently being tested in clinical trials (NCT04697940, NCT04850560 and NCT04553393). Thus, epigenetic regulation had the potential to become an important approach to reverse CAR T cell exhaustion in the future.

### Transcriptomic regulation of CAR T cell exhaustion

Multiple genes and transcription factors were involved in the regulatory network of CAR T cell exhaustion. One of the most important was the transcription factor TCF1, it was a key regulator of T cell differentiation. TCF1-positive CAR T cells exhibited a less exhaustion level and enhanced persistence *in vitro* and *in vivo* (Wu et al., 2016; Zheng et al., 2021). The other key transcription factors were the nuclear factor of activated T cells (NFAT) family and NR4A nuclear receptor family (NR4A1, NR4A2 and NR4A3). They played an important role in the immune homeostasis, including maintaining the development of regulatory T cells, regulating the activation of T cells, controlling the survival of B cells and T cells when encountered antigen (Macian, 2005; Sekiya et al., 2013; Hiwa et al., 2022). However, their strong activity and redundant effects were thought to be related to T cell exhaustion. It was reported that the NFAT/NR4A axis cooperatively controlled the expression of inhibitory receptors including PD1, TIM3 and LAG3. Deleting all three NR4A transcription factors could enhance the anti-tumor effect in solid tumor models (Table 1) (Chen et al., 2019). And meanwhile, Seo and colleagues observed that the transcription factors TOX and TOX2 were also downstream targets of NFAT to prompt CAR T cell exhaustion. Disruption the expression and activity of TOX and TOX2 in CAR T cells promoted tumor regression in tumor-bearing mouse models (Seo et al., 2019). Another NFAT partner, transcription factor AP1, was also involved in the regulation of CAR T cell exhaustion. Lynn et al. described that there was an epigenetic dysregulation of AP1 in exhausted CAR T cells using an exhaustion cell model with tonic CAR signaling, they proposed that this may be associated with the immunoregulatory AP-1/IRF transcriptional complexes (Lynn et al., 2019). In addition, some genes that may regulate CAR T cell exhaustion had been gradually uncovered through genetic screening. For example, one study identified that MAP4K1 gene (encoding HPK1) was highly correlated with the expression of inhibitory receptors in a variety of cancer through database analysis, the HPK1-NFκB-Blimp1 axis may mediate CAR T cell exhaustion. (Si et al., 2020). More recently, Wang et al. screened the whole genome of glioblastoma stem cells and CAR T cells to obtain the key molecules that determine the cytotoxicity of CAR T cells. They found that genes TLE2 and IKZF2 had a suppressive effect on CAR T cells. Intervention to down-regulate the expression of these two genes could inhibit CAR T cell exhaustion and enhance function in glioblastoma mouse models (Wang D. et al., 2021a). Therefore, with the clarity of genetic changes, the intricate regulation network behind CAR T cell exhaustion gradually emerged. Although the intervention of these transcription factors and genes was still confined to *in vitro* and preclinical stage currently, these regulators provided attractive drug targets for inhibiting CAR T cell exhaustion and improving immunotherapy response. Interventions against these key factors were expected to enter clinical applications soon.

## Conclusion

The mechanisms of CAR T cell exhaustion are extraordinarily complex and need to be explored in depth. First, inappropriate CAR T cell structure could induce ligand-independent tonic signaling and thus leading to CAR T cell exhaustion. Then, the cytokines and the duration of *in vitro* expansion also affect CAR T cell exhaustion. More importantly, there are abundant immunosuppressive factors in the TME. Hence, these multifaceted attacks make it difficult to reverse CAR T cell exhaustion. At present, PD1 and CTLA4 inhibitors are the dominant agents to combat CAR T cell exhaustion in clinical practice. However, the experience using immune checkpoint inhibitors with CAR T cells is limited. Besides, exhausted CAR T cells have epigenetic remodeling as well as transcriptomic abnormalities that increase the difficulty to intervene. Therefore, designing CAR T cells that can resist exhaustion or targeting these exhaustion-inducing factors may offer the potential to improve the efficacy of CAR T cells.
